# Spatial and sex-specific dissection of the *Anopheles gambiae *midgut transcriptome

**DOI:** 10.1186/1471-2164-8-37

**Published:** 2007-01-29

**Authors:** Emma Warr, Ruth Aguilar, Yuemei Dong, Vassiliki Mahairaki, George Dimopoulos

**Affiliations:** 1 W. Harry Feinstone Department of Molecular Microbiology and Immunology, Bloomberg School of Public Health, Johns Hopkins University, 615 N. Wolfe Street, Baltimore, MD 21205-2179, USA

## Abstract

**Background:**

The midgut of hematophagous insects, such as disease transmitting mosquitoes, carries out a variety of essential functions that mostly relate to blood feeding. The midgut of the female malaria vector mosquito *Anopheles gambiae *is a major site of interactions between the parasite and the vector. Distinct compartments and cell types of the midgut tissue carry out specific functions and vector borne pathogens interact and infect different parts of the midgut.

**Results:**

A microarray based global gene expression approach was used to compare transcript abundance in the four major female midgut compartments (cardia, anterior, anterior part of posterior and posterior part of posterior midgut) and between the male and female *Anopheles gambiae *midgut. Major differences between the female and male midgut gene expression relate to digestive processes and immunity. Each compartment has a distinct gene function profile with the posterior midgut expressing digestive enzyme genes and the cardia and anterior midgut expressing high levels of antimicrobial peptide and other immune gene transcripts. Interestingly, the cardia expressed several known anti-*Plasmodium *factors. A parallel peptidomic analysis of the cardia identified known mosquito antimicrobial peptides as well as several putative short secreted peptides that are likely to represent novel antimicrobial factors.

**Conclusion:**

The *A. gambiae *sex specific midgut and female midgut compartment specific transcriptomes correlates with their known functions. The significantly greater functional diversity of the female midgut relate to hematophagy that is associated with digestion and nutrition uptake as well as exposes it to a variety of pathogens, and promotes growth of its endogenous microbial flora. The strikingly high proportion of immunity related factors in the cardia tissue most likely serves the function to increase sterility of ingested sugar and blood. A detailed characterization of  the functional specificities of the female mosquito midgut and its  various compartments can greatly contribute to our understanding of its  role in disease transmission and generate the necessary tools for the  development of malaria control strategies.

## Background

The midgut of the female *Anopheles *mosquito is responsible for the digestion of the blood meal and is the entry point and site of extensive interaction with disease-causing pathogens. Female mosquitoes are capable of transmitting a variety of pathogens and as such the midgut has become an important focus for studies that aim at understanding the transmission biology of vector-borne diseases [1-10].

**Figure 1:**
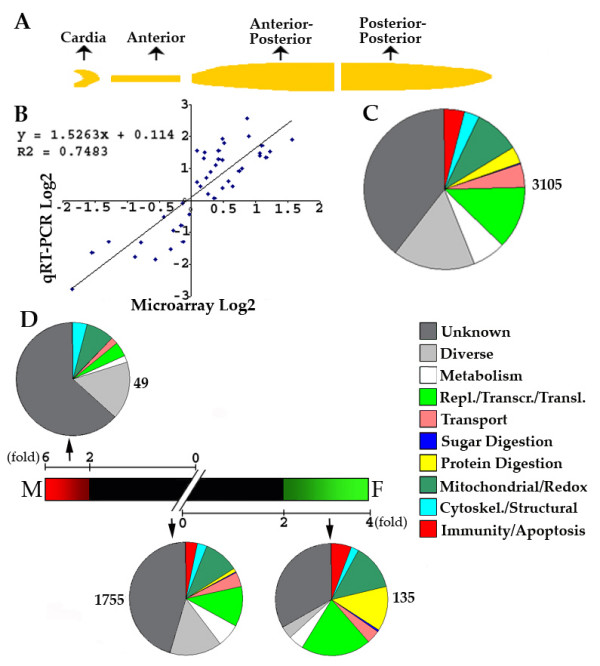
**A**. Compartmentalization of the *A. gambiae *midgut in 4 parts: cardia, anterior, anterior-posterior and posterior-posterior. **B**: Validation of microarray-assayed gene expression with real-time quantitative RT-PCR. Expression data (log2 ratio) of 11 genes from four midgut assays (male versus female; anterior midgut versus whole female midgut, anterior part of posterior midgut versus whole female midgut; cardia versus posterior midgut) obtained from microarray analysis were plotted against the corresponding expression values obtained with real time RT-PCR from the mean of the three replicates of each experiment. The best-fit linear-regression analysis (R^2 ^= 0.7483) and the slope of the regression line (m = 1.5263) demonstrate a high degree of correlation of the magnitude of regulation between the two assays. **C**: Functional gene class distribution of the putative total midgut transcriptome as predicted by the pooled transcriptomes of female and male midguts, and all individual midgut compartments that were assayed in this study. **D**: Comparison of the functional class distribution of transcripts in the midguts of female and male mosquitoes. Transcripts enriched by greater than a 2-fold in the female and male midguts are represented by the green and red sections, respectively. Transcripts with similar abundance in midguts of both sexes are represented by the black section. The functional gene class distribution within each group is indicated by the pie charts.

A variety of studies, including microarray-based gene expression analyses, have addressed the effect of infection or blood-meal ingestion on the female mosquito. These studies have established the immune responsive capacity of the midgut and provide detailed information about a variety of biological processes that are regulated upon blood feeding [1-3,5-12]. However, these studies have largely focused on the overall mosquito midgut transcriptome, ignoring the spatial specialization of different functions in the various compartments of this tissue (Figure 1a).

Adult female mosquitoes feed on  both blood and sugar, and the storage and processing of these two foods  are spatially different. The ingested sugar is first stored in the  ventral diverticulum (crop) and then delivered to the midgut. Its digestion and absorption occurs within both the anterior and posterior midgut [13,14]. The alpha-glucosidase activity responsible for sugar digestion is detected in both compartments, although regulation does differ between the two; the enzyme activity is constitutively expressed in the anterior midgut, and blood-feeding does not influence its levels [15]. In contrast to sugar digestion, the blood meal is exclusively digested and absorbed in the posterior region [13,16]. Aminopeptidases and glucosidases are also detected in the posterior region, where their levels rise after blood-feeding. Digestion of the protein content of blood is mainly carried out by serine proteases such as trypsins and chymotrypsins that display complex patterns of regulation in relation to blood feeding. Trypsin activity is limited to the posterior region of the midgut [15,17-19]. The majority of the cells in the posterior midgut are digestive epithelial cells, which produce and secrete the enzymes needed for blood-meal digestion [14]. Regenerative and endocrine cells are also present in the posterior midgut, although their function is currently unknown [14]. In addition to their essential  role in blood-meal digestion, trypsins have also been shown to be  required for the activation of an ookinete-secreted chitinase, which  enables the parasite to penetrate the peritrophic matrix of the mosquito  midgut [20-22]. A variety of transporters responsible for maintaining osmotic balance and nutritient uptake are also expressed in the midgut and are regulated upon feeding [9].

The peritrophic matrix of mosquitoes is a semi-permeable chitinous matrix that lines the digestive tract and thereby acts as a barrier to direct contact with the blood meal and to microbial and parasitic infections [23,24]. The peritrophic matrix is synthesized by midgut epithelial cells in direct response to the ingestion of a blood meal. The peritrophic matrix precursors are stored in secretory vesicles of midgut epithelial cells, and the secretion of these proteins is triggered by blood-feeding, releasing the vesicle contents into the lumen of the mosquito midgut [25]. The malarial parasite *Plasmodium *has to cross the peritrophic matrix before it can invade the midgut epithelium and, as such, the peritrophic matrix and the genes involved in its formation have therefore become a focus for studies that aim to block parasite transmission [25-28].

In *Plasmodium*-infected mosquitoes, oocysts are primarily found in the very posterior region of the midgut [29]. The reason(s) for this spatial distribution is still unclear. An early suggestion was that the parasites preferentially invade vesicular ATPase (V-ATPase)-overexpressing cells that are located at the very posterior region of the midgut [29,30]. More recently, it has been shown that not all *Plasmodium *species specifically invade this particular type of cell [31].

The close association between transcript abundance and gene function makes it possible to infer biological function and physiological conditions from gene expression profiles. We have performed a global microarray-based gene expression analysis to study the midgut sex- and compartment-(cardia, anterior, anterior-posterior and posterior-posterior) specific transcriptomes. In conjunction with this analysis, we have utilized a peptidomic approach to identify putative short secreted peptides that are enriched in the cardia and anterior compartment, where the majority of the mosquito's antimicrobial peptides are found to be produced. The elucidation of these spatial profiles (both transcriptomic and peptidomic) will provide a broadened basis for understanding the physiological significance and functional attributes of the *A. gambiae *midgut, and may lead to the identification of components that can be used to develop novel strategies to disrupt pathogen transmission.

## Results and discussion

The *Anopheles gambiae *sex-specific midgut transcriptomes and the female midgut compartment-(cardia, anterior, anterior-posterior and posterior-posterior) specific transcriptomes were characterized using microarrays that represented the entire mosquito transcriptome [4]. For the midgut compartment-specific transcription assays, labeled samples made from RNA from each compartment were hybridized against a labeled sample made from RNA of the entire midgut. Genes that exhibited differential expression of 2-fold or greater between the compared samples were considered to be significantly enriched in either one of the samples. Expression data generated by the microarray analysis were validated by independently determining the regulation of 11 genes with real-time quantitative PCR (QRT-PCR) (Figure 1b).

### *Anopheles gambiae *sex-specific midgut transcriptome

The 4-day-old mosquito midgut expressed 3105 genes in total (estimate based on the threshold hybridization signals of labeled samples made from the female and male whole midgut and the female midgut compartments as described in the methods section) (Figure 1c). I direct comparison of transcript abundance between the female and male midguts identified 135 transcripts that were specifically enriched in the female midgut and 49 transcripts that were specifically enriched in the male midgut (Figure 1c, d) (Additional file 1). The remaining 1755 transcripts were present at equal levels in both the male and female midguts, and they represented a variety of functional classes. The most prominent of these classes were replication/transcription/translation (R/T/T), metabolism, and redox/mitochondrial (Figure 1d). A total of 58 immunity and apoptosis -related transcripts were expressed in both female and male midguts, including three known anti-microbial peptides, gambicin (EN013255), cecropin CEC3/CECB (EN011995) and cecropin SEC2/SECC (EN011963) (Additional file 1). Thirty transcripts encoding putative pattern recognition receptors were also highly expressed in the midguts of both sexes. These included members of the C-type lectin, ML2-like family (AGMDL), fibrinogen domain immunolectin (FBN), Galectin, Gram-negative bacteria binding protein (GNBP), peptidoglycan recognition protein (PGRP), scavenger receptor, leucin rich repeat domain gene family (LRRD), and thioester containing protein (TEP) families (Additional file 1). A further eight transcripts are involved in putative immune amplification and signalling pathways: REL2 (EN020234), TOLL6 (EN008963), two immune-related serine proteases CLIPD6 (EN026417) and SP22D (EN021796), and four serpins (EN010545, EN024799, EN024759, EN024786) (Additional file 1).

The female midgut-specific genes displayed a significantly greater functional diversity than did the male midgut-specific genes, which were mostly involved in housekeeping processes. The functional differences between the transcriptomes of the male and female midguts were mainly related to immunity and blood digestion (Figure 1d). A total of 17 transcripts involved in proteolysis including several blood digestive enzyme genes such as carboxypeptidases (EN021052, EN020553), and trypsins 3, 4, 5 and 7 (EN006721, EN018384, EN018317, EN018316). A previous report also showed that these members of the trypsin family are constitutively expressed in unfed female mosquitoes while two (TRY-1 and -2) are blood-meal-inducible [19]. In contrast, the male midgut did not display specific expression of single proteolytic enzyme transcript. Two peritrophin 1 precursors (EN013392, EN023523) were also found to be highly expressed in the female gut (Additional file 1). In contrast to the female midgut, the male gut was not enriched in transcripts that are known to play a role in blood digestion (Figure 1d).

Eight transcripts pertaining to immunity-related functions were enriched in the midguts of female mosquitoes; these included two members of the FBN family (EN019563, EN022867), serpin 15 (EN024710), prophenoloxidase 9 (EN010740), a lysozyme (EN025070), an immunoglobulin-like precursor (EN027057) and nitric oxide synthase (EN019316) (Additional file 1). In contrast, the male midguts did not exhibit specific expression of any immune genes (Additional file 1).

### Female midgut compartment-specific transcriptomes

**Figure 2:**
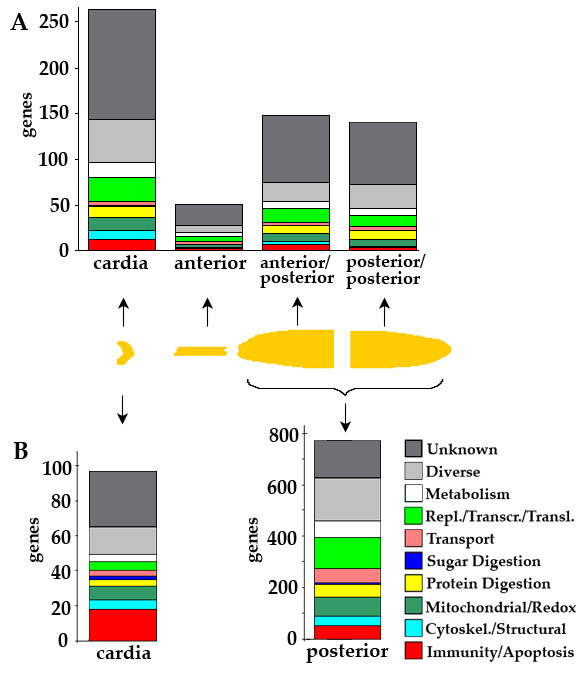
**A**: Functional class distribution of transcriptomes enriched in any of the assayed compartments by greater than a 2-fold, compared to the whole female midgut. **B**: Functional class distribution of transcriptomes enriched in the cardia and the posterior midgut compartments by equal or greater than a 2-fold upon comparison between these two samples.

To investigate the female midgut compartment-specific transcriptomes, the gene-specific mRNA abundance of each compartment (cardia, anterior, anterior-posterior and posterior-posterior) was compared to that of the whole female mosquito midgut (Figure 2a). The relatively small number of posterior part-enriched (anterior-posterior and posterior-posterior) genes identified in these assays is attributed to the significantly larger number of cells contained in the posterior midgut (and hence larger contribution of this compartment towards the total RNA of the midgut) and the quite similar cell makeup of the two parts in conjunction with our hybridization strategy that was based on a hybridization between a posterior compartment-specific labeled sample against the whole midgut sample, which also included the same posterior compartment transcripts. For this reason a second set of hybridizations was also performed to directly compare gene expression in the cardia and posterior midgut (Figure 2b) (Additional file 1). These assays identified a  significantly larger number of posterior midgut-enriched transcripts.

The cardia tissue displayed specific expression of 264 transcripts when compared to the whole female mosquito gut (Figure 2a) (Additional file 1). Direct comparison of gene expression between the cardia and the posterior midgut identified 97 cardia-specific transcripts, of which 18.5% (18 transcripts) belonged to the immunity class (Figure 2b) (Additional file 1). All of the known mosquito anti-microbial peptides cecropin CEC3/CECB (EN011995), cecropin CEC2/CECC (EN011963), gambicin (EN013255), and defensin 1 (EN015621) were all significantly enriched in the cardia when compared to the other compartments (Additional file 1). Two lysozyme transcripts (EN018439, EN018395) were also highly enriched in the cardia. Expression of Gambicin and defensin has previously been localized to the cardia [17], and both these genes have been shown to be induced in the midgut upon invasion by malarial parasites [1,2]. Defensin has been shown to be expressed by the anterior midgut of another hematophagous insect, *Stomoxys calcitrans *[32]. The cardia showed highly enriched expression of seven anti-*Plasmodium *factors including Tep1 (EN016857), LRRD7 (EN021822), FBN8 (EN011252), FBN9 (EN011248), SP CLIP1 (EN020158) and Gambicin (EN013255) [4,33,34](Dimopoulos lab, unpublished data).

The anterior compartment, which mainly serves as a tube to allow the blood to enter the posterior section, displayed the smallest number of enriched genes: only 51 transcripts of varying functions (Figure 2a) (Additional file 1). Three of these genes encoded proteins involved in immune defense: TEP3 (EN016283), TEP16 (EN021282), and PGRPLB (EN013948) (Additional file 1).

The anterior part of the  posterior midgut displayed enriched expression of 148 transcripts with  quite diverse functions (Figure 2a). Transcripts that play a role in proteolysis, basic metabolism, and immunity represented a significant portion of these transcripts (Figure 2b, Additional file 1). Among 7 immunity-related transcripts were 3 anti-*Plasmodium *factors, TEP1 (EN016857), NOS (EN019316) and FBN7 (EN008282) [3,33](Dimopoulos lab, unpublished data). The most posterior compartment, the posterior part of the posterior midgut, was enriched for 142 transcripts that had at least a two-fold higher expression in this compartment than in the whole female gut and had a functional class distribution with similar proportions to that of the anterior part of the posterior midgut, while the actual genes are different (Figure 2a, b). Only 4 transcripts encoding potential immune-related factors were more specifically expressed in this compartment and none of those represented known anti-*Plasmodium *factors, suggesting that the anterior parts of the midgut is potentially more active in anti-*Plasmodium *gene expression (Additional file 1).

A direct comparison of gene expression between the cardia and the posterior midgut identified 867 differentially expressed genes, of which 770 were enriched in the posterior midgut (Figure 2b). A major functional group of the posterior midgut was represented by 49 transcripts encoding proteolytic enzymes; many of those are know to be involved in blood digestion. (Additional file 1). It has previously been shown that several blood digestive proteases are expressed prior to feeding in order to be immediately present upon blood ingestion [17-19]. Several of these proteolytic enzymes may be involved in diverse processes such as signal transduction, extracellular matrix processing and immunity. The posterior midgut displayed elevated expression of 52 transcripts involved in immunity (6.7% of total posterior midgut specific genes) (Figure 2b, Additional file 1). As many as 34 of these putative immune genes encoded putative pattern recognition receptors, including two *Plasmodium *protective c-type lectins CTL4 (EN021138) and CTLMA2 (EN020902) (Additional file 1).

### Peptidomic analysis of peptides within the cardia and posterior compartments

The transcription analyses indicated the cardia as a major site of antimicrobial gene expression. In order to identify potential  novel antimicrobial peptides, we employed a pepdidomic approach using  2-dimensional liquid chromatography tandem mass spectrometry (LC/MS/MS)  to screen for putative secreted short peptides that are enriched in the  cardia compartments of the female mosquito midgut. The selected approach allowed the sample to be fractionated into several reversed-phase runs, giving better coverage by virtue of less complexity in any given fraction. This was, however, not a comprehensive analysis of all the proteins/peptides that are present in the cardia since a protein extraction method that is biased towards short peptides was used.

A total of 94 proteins were identified in the cardia peptide extract (Additional file 2). The peptidomic analysis was overlaid with the transcriptomic data to identify those gene products that have corresponding data: 19.6% of the identified peptides had corresponding transcript expression data (Table 1). The relatively small overlap between transcriptome and peptidome data is most likely attributed to differences between mRNA and protein turnover rates and abundance that therefore does not permit detection by either one of the two methods. Ten peptides were identified in the cardia that had both a length of less than 133 amino acids and a signal peptide, as predicted by SignalP 3.0 server (Technical University of Denmark DTU) (Table 2). Seven of these peptides are annotated in Ensembl and include three of the known mosquito anti-microbial peptides (cecropins CEC2, CEC3, and defensin) (Table 2). A heat shock protein (ENP011747), a cuticle protein (ENP014823), and a salivary gland transcript of unknown function CE5 (ENP012315) were also among these putative short secreted peptides (Table 2). BLAST analysis of the CE5 amino acid sequence revealed that it has a 52% identity to an anti-thrombin protein found in the salivary gland of *Anopheles stephensi *[35]. Cuticle proteins have previously been linked to immune defense reactions in female *A. g*ambiae [12]. The remaining three putative short secreted peptides (ENP014492, ENP030767, ENP018638) are of unknown function.

Three histone proteins (ENP000003, ENP008313, ENP024235) were also present in the cardia (Additional file 2); a protein family that has been previously been linked to immunity [12,36,37]. Two peptides (ENP021938, ENP029123) of 187 and 175 amino acids in length, respectively, had a predicted signal peptide and contained a pyrokinin domain (Additional file 2). Pyrokinins are insect neurohormones that are involved in signalling pathways and mediate muscle contractile activity [38]. The presence of such enzymes in the unfed mosquito midgut is of particular interest since the *Opisina arenosella *pyrokinins have been found to possess inhibitory effect on the digestive enzyme release in the midgut [39].

## Conclusion

In this work we have analyzed the *Anopheles gambiae *sex-specific midgut transcriptome and dissected the female midgut transcriptome into spatially specific compartments.

Despite the broad functional differences between the female and male midgut, only a limited degree of differential expression was detected (Figure 1b, Additional file 1). A similar observation was made in a proteomic study that also found very few differences in protein expression between male and unfed female midguts: ten male and eight female-specific proteins [40]. A further ten female-specific proteins were identified after blood-feeding in the same study. The unfed state of the midgut tissues used for our assays is probably responsible for our inability to identify these sex-specific transcripts, which are mainly functionally related to blood digestion and have been described elsewhere [8,9].

The two major differences in functional class between the transcriptomes of male and female mosquito midguts mainly reflect the exclusive hematophagous nature of the female mosquito that also exposes it to a greater spectrum and level of microbes [41]. These gene classes, related largely to blood digestion and immunity, and are highly enriched in the female midgut (Figure 1b, Additional file 1).

Our analysis revealed that the different compartments of the female mosquito midgut display distinctive gene expression patterns that correlate with their specific functions. The posterior compartment is the site of blood-meal digestion; it expressed a variety of digestive enzymes implicated in both sugar and protein digestion, whereas the more anterior compartments appeared to be more specialized in sugar digestion (Figure 2); differences between the transcriptomes of the posterior and cardia compartments were mainly related to proteolysis/digestion, metabolism, and immunity (Figure 2). Interestingly, the anti-*Plasmodium *factors Tep1 and nitric oxide synthase displayed elevated expression in the anterior part of the posterior midgut [3,33]. This spatial expression pattern, taken together with the highly elevated expression of another seven anti-*Plasmodium *factors in the cardia and anterior compartment, could partly explain the higher *Plasmodium *infection levels in the posterior part of the posterior midgut. Interestingly, both *Plasmodium *protective c-type lectins, CTLMA2 and CTL4 were highly expressed in the posterior section [42]. Additional anti-*Plasmodium*  factors are likely to be expressed in the midgut tissue but not  identified in the present study because of the non-infected state of the  studied mosquitoes. Immune gene expression patterns also displayed qualitative differences between the cardia and posterior compartment that were principally related to the exclusive expression of antimicrobial peptide genes and lysozyme precursors in the cardia. The elevated presence of these immune factors in the cardia is likely to serve a mechanism that maintains the sugar rich anterior midgut sterile, and also allow efficient secretion and distribution in the blood meal while it is ingested and passes through the cardia and anterior midgut compartment. Antimicrobial peptide sequences have diverged rapidly during evolution and only a few share extensive homologous across species. One common feature of most antimicrobial peptides is their short and secreted nature. Based on our findings, the cardia can be expected to produce a variety of novel mosquito-specific antimicrobial peptides and has therefore a potential to serve as a useful source for their identification and further study. Four cardia-enriched transcripts (EN004315, EN014362, EN009630, EN016194) and two peptides identified in the cardia proteome (ENP014492, ENP030767) encode such short and secreted peptides (Additional file 1, Table 2). One of these (EN009630) has recently been identified as a putative short immune peptide that is strongly induced by *P. falciparum *invasion of the midgut, although silencing of this gene was found to have no effect on *Plasmodium *development [4].

The *Anopheles *midgut is the major site of contact and interaction with the *Plasmodium *parasite. Malaria control strategies based on transgenic expression of anti-*Plasmodium *factors that target the parasite in the mosquito would require both effector genes with plasmodiocidal activity, and tissue- and stage-specific promoters [27,43,44]. Targeting the *Plasmodium *parasite in the midgut would require spatial specificity of anti-*Plasmodium *gene expression depending on the targeted parasite stage. For instance, a cardia specific promoter may be more appropriate to drive expression of an anti-*Plasmodium *factor that target gametocytes, zygotes and ookinetes in the midgut lumen as it potentially would allow the factor to blend into the ingested blood meal. An anti-*Plasmodium *factor that kills ookinete stages in the midgut epithelium would be more effective against the parasite if expressed in the posterior compartment which is invaded. This comprehensive study and other studies have the potential to provide such promoters and anti-*Plasmodium *factors.

## Methods

### Mosquito rearing and sample collection

*A. gambiae *Keele strain mosquitoes were raised at 27°C and 70% humidity, and adults were maintained on a 10% sucrose solution. Midguts from 4-day-old adult mosquitoes were dissected on ice in PBS (0.6 mM MgCl_2_, 4 mM KCl, 1.8 mM NaHCO_3_, 150 mM NaCl, 25 mM HEPES, 1.7 mM CaCl_2_, pH 7) and immediately frozen with dry ice. Total RNA was extracted with either the Mini RNA isolation kit (Zymo Research, Orange, CA) or the RNeasy mini kit (Qiagen, Valencia, CA) according to the manufacturer's instructions.

### Microarray assays

Double-stranded cDNA primed with an oligo d(T)-T7 promoter, produced from total RNA (2 μg), was used to synthesize complementary RNA (cRNA) with incorporated Cy-3-dUTP and Cy-5-dUTP fluorescent nucleotides, using the Agilent Low RNA input Fluorescent Linear Amplification Kit (Agilent Technologies, Palo Alto, CA). Unincorporated dye-labeled nucleotides were removed with the Qiagen PCR purification kit (Qiagen, Chatsworth, CA). A 60-mer oligonucleotide microarray representing the entire *A. gambiae *transcriptome was used for these assays [4]. To compare the transcriptomes of the male and female midgut, Cy-5-labeled cDNA targets made from the midgut RNA of male mosquitoes were hybridized against a Cy-3-labeled reference probe made from the midgut RNA of female mosquitoes. To assay the transcriptomes of the individual compartments of the gut, Cy-5-labeled cDNA targets made from the RNA of female midgut compartments (cardia, anterior, anterior-posterior, and posterior-posterior) were hybridized against a Cy-3-labeled reference probe made from the midgut RNA of whole female midguts. To compare the gene expression of the cardia and the posterior compartments of the midgut, Cy-5-labeled cDNA targets made from the RNA of the female cardia midgut compartment were hybridized against a Cy-3-labeled reference probe made from the RNA of the female posterior midgut. Three biological replica assays were performed for each experiment.

### Data analysis

Spot intensities were measured with a GenePix 4200AL autoloader scanner (Axon Instruments). Images were inspected manually using GenePix Pro 6.0 software (Axon Instruments), and any spots that were covered with hybridization artefacts were removed and were not included in the further analysis. The TIGR MIDAS software was used to filter the data set using a hybridization signal cut-off of 100 units to remove low intensity spots from the analysis, and Loc-Fit normalization (LOWESS) was performed for all data sets independently to adjust for dye-specific biases. The normalized Cy5/Cy3 ratios from replicate assays were subjected to *t*-tests at a significance level of p ≤ 0.05 using TIGR MIDAS and MeV software. The replicate Cy5/Cy3 ratios for each transcript were averaged using the GEPAS (Gene Expression Pattern Analysis Suite v1.1, available free online ) after the data set had been processed and filtered. Microarray data sets have been submitted to the GEO-NCBI with the following accession numbers: GSM146402, GSM146403, GSM146404, GSM146405, GSM146406, GSM146407, GSM146408, GSM146409, GSM146410, GSM146411, GSM146412, GSM146413, GSM146414, GSM146415, GSM146416, GSM146417, GSM146418, GSM146419.

### Real time quantitative PCR

RNA samples were treated with Turbo DNase (Ambion) and reverse-transcribed using Superscript III (Invitrogen) with random hexamers. Real-time quantification was performed using the QuantiTect SYBR Green PCR Kit (Qiagen) and ABI Detection System ABI Prism 7000. All PCR reactions were performed in triplicate. Specificity of the PCR reactions was assessed by analysis of melting curves for each data point. The ribosomal protein S7 gene was used for normalization of cDNA templates. Primer sequences are listed in table Additional file 4.

### 2-dimensional chromatography and SCX-LC/MS/MS LTQ analysis

Midguts were dissected on a chilled platform, and the cardia and posterior compartments were collected separately on dry ice. The tissue was homogenized in methanol/water/acetic acid (87:8:5 v:v:v), then centrifuged (8000 g for 10 min at 4°C). Peptides present in the supernatant were collected, and the pellet was re-homogenized and centrifuged. The combined supernatants were lyophilized and stored at -20°C until required. The 2-dimensional chromatography method used employs a strong cation exchange separation (SCX) (PolyLC 300 um × 100 mm 200A 5 um) in the first dimension, followed by reversed-phase chromatography in the second dimension. An additional reversed-phase cleanup step was used to rid the sample of any residual salts that might affect retention on the SCX column. Samples were bound and eluted from a C18 cartridge (Sep-Pak, Waters), elute, and dried down before being re-suspended in 24 μl of 0.1% formic acid. Each sample was then loaded onto the SCX column as three 8-μl aliquots, back-to-back. Three 8-μl salt elution steps were performed with each fraction at 0 mM, 20 mM, 30 mM, 40 mM, 125 mM, 250 mM and 1000 mM KCl in 0.1% formic acid. Each salt fraction was trapped and eluted onto the reversed-phase column (a C18 7-μM column packed with YMC ODS-AQ 5 μm particle size, 120A pore size). The DBParser program was used to collate the database search results and to remove redundant proteins and peptide hits that did not meet the data-set significance threshold. The criteria for protein identification were based on more than one peptide hit with the individual peptide scores set above the data-set threshold.

## Abbreviations

ENSEMBL gene IDs have been abbreviated by omitting the "NSANG00000" portion.

ENSEMBL protein IDs have been abbreviated by replacing the "SANGP00000" portion with P.

## Authors' contributions

EW, RA and YD dissected the mosquito midgut samples and prepared RNA. EW performed microarray analyses and real time PCR assays. VM contributed with preliminary data sets of the mosquito midgut transcriptome. EW and GD wrote the manuscript. All authors read and approved the final manuscript. All authors read and approved the final manuscript.

## Supplementary Material

Additional File 1 The midgut transcriptome. Processed microarray generated comparative gene expression ratios for male versus female comparisons, female midgut compartment (cardia, anterior, anterior-posterior, posterior-posterior) versus whole female midgut, and female midgut cardia compartment versus posterior midgut. Click here for file

Additional File 2 The cardia peptidome. Peptidomic analysis of the cardia tissue identified 94 putative proteins. Transcript (ENSANGT) gene (ENSANGG) and protein (ENSANGP) identifiers are indicated. The length (AA), predicted signal peptide (SP) and putative protein family/homology are indicated. Click here for file

Additional File 4 Real time RT-PCR primers. DNA sequences of primers used for real time RT-PCR analyses. Click here for file

Additional File 3 Microarray data validation with real time RT-PCR. Validation of microarray-assayed gene expression with real-time quantitative RT-PCR. Expression data (log2 ratio) of 11 genes from four midgut assays (male versus female; versus whole female midgut, anterior-posterior versus whole female midgut; cardia versus posterior midgut) obtained from microarray analysis were validated with 3 replica real time RT-PCR assays and presented in figure 1A. m, male gut; f, female gut; c, cardia; p, posterior; a, anterior; a/p, anterior-posterior. Click here for file

## Figures and Tables

**Table 1:** Peptidomic analysis of the cardia compartment.

**ENSANGP**	**ENSANGT**	**PROTEIN FAMILY**	**TRANSCRIPTION (C)/(P)**
ENP007677	EN007677	AMBIGUOUS	0.712
ENP008729	EN008729	28S RIBOSOMAL	0.54
ENP010425	EN010425	AMBIGUOUS	0.751
ENP010332	EN010332	REGUCALCIN RC SENESCENCE MARKER	2.159
ENP011963	EN011963	CECROPIN CEC2	2.208
ENP011995	EN011995	CECROPIN CEC3	2.967
ENP012315	EN012315	CE5 PROTEIN	2.307
ENP015570	EN015570	ADAPTIN	0.626
ENP015621	EN015621	DEFENSIN DEF1	2.328
ENP017570	EN017570	MITOCHONDRIAL TRANSLOCASE SUBUNIT	0.389
ENP019649	EN019649	GLUCOSAMINE ACETYLTRANSFERASE	0.728
ENP020396	EN020396	SUCCINYL COA LIGASE	0.562
ENP020907	EN020907	CALCIUM BINDING CEX 1	0.76
ENP021422	EN021422	SOLUTE CARRIER FAMILY MEMBER	0.686
ENP021618	EN021618	FAM61A	0.661
ENP021938	EN021938	PYROKININ	0.664
ENP023148	EN024839	CALCIUM BINDING 39 MO25	0.644
ENP027189	EN025727	CLIPA4	0.414
ENP029123	EN026942	UNKNOWN	1.698
ENP010310	EN010310	CYTOCHROME C OXIDASE	0.564
ENP012554	EN012554	60S RIBOSOMAL L23A	0.641
ENP014265	EN014265	60S RIBOSOMAL L28	0.526
ENP017618	EN017618	40S RIBOSOMAL S25	0.459
ENP018502	EN018502	VACUOLAR ATP SYNTHASE	0.475
ENP018638	EN018638	UNKNOWN	2.434
ENP018909	EN018909	60S RIBOSOMAL L30	0.469
ENP019335	EN019335	UNKNOWN	0.522
ENP020553	EN020553	CARBOXYPEPTIDASE	0.558

Gene products identified from the peptidomic analysis and the corresponding transcript expression data, as identified from the microarray analysis, for the cardia and posterior compartments of the midguts of female *A. gambiae*. With regard to the transcriptomic analysis, a -fold ratio of ≥2 denotes those transcripts that had a significantly higher expression in the cardia (C) as compared to the posterior (P), and a -fold ratio of ≤0.5 denotes those transcripts that had a significantly higher expression in the posterior compartment (as compared to the cardia). A -fold ratio between 0.5 and 2 denotes those transcripts that did not exhibit differential expression between the cardia and posterior compartments of the female midgut.

**Table 2:** Antimicrobial peptides and putative short, secreted peptides present in the cardia compartment.

**ENSANGP**	**PROTEIN FAMILY**	**LENGTH (AA)**	**SP CLEAVAGE SITE**
ENP011747	10 KDA HEAT SHOCK PROTEIN	101	21–22
ENP011963	CECROPIN CEC2	58	23–24
ENP011995	CECROPIN CEC3	60	25–26
ENP012315	CE5 PROTEIN	103	21–22
ENP014492	UNKNOWN	82	20–21
ENP014823	CUTICLE	133	16–17
ENP015621	DEFENSIN DEF1	133	46–47
ENP030767	UNKNOWN	117	21–22
ENP010310	CYTOCHROME C OXIDASE	82	26–27
ENP018638	UNKNOWN	51	24–25

Putative short, secreted peptides present in the cardia compartment of the female mosquito midgut as detected by two-dimensional chromatography and SCX- LC/MS/MS LTQ. Only those peptides that have a length equal or shorter than the mosquito anti-microbial peptides defensin (≤133 amino acids) are presented here. The signal peptide (SP) cleavage site is indicated.
