# Supplementary material for: Spatial and sex-specific dissection of the Anopheles gambiae midgut transcriptome
Source: BMC Genomics. 2007 Jan 29;8:37. doi: 10.1186/1471-2164-8-37 (PMC1804276; doi:10.1186/1471-2164-8-37)
Supplement: Additional File 4 — Real time RT-PCR primers. DNA sequences of primers used for real time RT-PCR analyses. [file 1471-2164-8-37-S4.pdf]

Additional file 4

Primers used for real time RT-PCR analyses:

Cecropin 3A: 5'-GTTTCGGGCGGCTAAGAA-3'

Cecropin 3B: 5'-AACAAACACGCACGCACA-3'

Defensin 1A: 5'-GAGAACTATCGGGCCAAGC-3'

Defensin 1B: 5'-TGCAATTTTCATCCCGATT-3'

Gambicin A: 5'-GGCTATCTCAACCGGAAGG-3'

Gambicin B: 5'-CGGCAGCCGTTGCGGATGCAATG-3'

PGRPLC1 A: 5'-TACTGCTGCTTCGCAACG-3'

PGRPLC1 B: 5'-ATCGACCAACGGAACAGC-3'

PPO9 A: 5'-AGTTCAACTTCTGCGGCTGT-3'

PPO9 B: 5'-AAGTTGGCCAAACTGTCCAC-3'

TEP15 A: 5'-ACATCTGCGAAAACGAGGAC-3'

TEP15 B: 5'-GCAACTGTACCATCCCTGCT-3'

LRD17594 A: 5'-GTTCGATTTTACCCCCGAGT-3'

LRD17594 B: 5'-GTACAATCCATGAGGTTTCGATC-3'

LRD15794 A: 5'-CGCCTTTCCCGTGCTGGAGG-3'

LRD15794 B: 5'-CGTCTTGATGTACTCGAGCAC-3'

BRP5241 A: 5'-CATTCCCCTGCTGAAGGATA-3'

BRP5241 B: 5'-GTTCACCGGTGACAGCGTGCTC-3'

TEP4 A: 5'-TGTTGATTGCACTGCTGGA-3'

TEP4 B: 5'-GGAGGGGCCGTCTTTCTGACCGC-3'

GNBPB3 A: 5'-TGGGGATGTGAGCGTACC-3'

GNBPB3 B: 5'-CGATTCCCATCCGTTCAG-3'

Kininogen A: 5'-TTTGTTGGCGATCTTTGTCA-3'

Kininogen B: 5'-CTGACCAGTCCGCCCGGAGACGC-3'
