# Supplementary material for: Spatial and sex-specific dissection of the Anopheles gambiae midgut transcriptome
Source: BMC Genomics. 2007 Jan 29;8:37. doi: 10.1186/1471-2164-8-37 (PMC1804276; doi:10.1186/1471-2164-8-37)
Supplement: Additional File 3 — Microarray data validation with real time RT-PCR. Validation of microarray-assayed gene expression with real-time quantitative RT-PCR. Expression data (log2 ratio) of 11 genes from four midgut assays (male versus female; versus whole female midgut, anterior-posterior versus whole female midgut; cardia versus posterior midgut) obtained from microarray analysis were validated with 3 replica real time RT-PCR assays and presented in figure 1A. m, male gut; f, female gut; c, cardia; p, posterior; a, anterior; a/p, anterior-posterior. [file 1471-2164-8-37-S3.pdf]

Additional file 3

Validation of microarray-assayed gene expression with real-time quantitative RT-PCR. Expression data (log2 ratio) of 11 genes from four midgut assays (male versus female; versus whole female midgut, anterior-posterior versus whole female midgut; cardia versus posterior midgut) obtained from microarray analysis were validated with 3 replica real time RT-PCR assays and presented in figure 1A. m, male gut; f, female gut; c, cardia; p, posterior; a, anterior; a/p, anterior-posterior.

Assay/gene	rep1	rep2	rep3	ave	SD	SE	LOG2 of average	array LOG2
m vs f								
Cecropin 3	**3.89**	**2.93**	**3.71**	3.51	0.51	0.36	**1.81**	0.51
c vs p								
Cecropin 3	**3.51**	**2.88**	**4.81**	3.73	0.98	0.70	**1.90**	1.565
a vs f								
Cecropin 3	**0.35**	**0.37**	**0.51**	0.41	0.09	0.06	**-1.29**	-0.13
a/p vs f								
Cecropin 3	**0.10**	**0.16**	**0.18**	0.15	0.04	0.03	**-2.77**	-1.862
m vs f								
Defensin 1	**1.54**	**1.45**	**1.69**	1.56	0.12	0.09	**0.64**	0.08775385
c vs p								
Defensin 1	**3.67**	**2.12**	**2.84**	2.88	0.78	0.55	**1.52**	1.218
a vs f								
Defensin 1	**0.41**	**0.37**	**0.26**	0.35	0.08	0.06	**-1.53**	-0.32
a/p vs f								
Defensin 1	**0.66**	**0.77**	**0.34**	0.59	0.22	0.16	**-0.76**	-0.16

m vs f								
Gambicin	**0.97**	**0.90**	**0.94**	0.94	0.04	0.02	**-0.09**	-0.144
c vs p								
Gambicin	**3.86**	**1.73**	**2.31**	2.63	1.10	0.78	**1.40**	1.063
a vs f								
Gambicin	**0.65**	**1.16**	**1.25**	1.02	0.32	0.23	**0.03**	-0.11
a/p vs f								
Gambicin	**0.76**	**0.69**	**0.65**	0.70	0.06	0.04	**-0.51**	-0.43
m vs f								
PGRPLC1	**0.86**	**0.77**	**0.60**	0.74	0.13	0.09	**-0.43**	-0.042
c vs p								
PGRPLC1	**2.57**	**2.08**	**3.69**	2.78	0.83	0.59	**1.47**	1.075
a vs f								
PGRPLC1	**1.78**	**2.22**	**2.02**	2.01	0.22	0.16	**1.00**	0.789
a/p vs f								
PGRPLC1	**1.16**	**0.94**	**1.05**	1.05	0.11	0.08	**0.07**	0.36
m vs f								
PPO9	**0.27**	**0.30**	**0.31**	0.29	0.02	0.01	**-1.77**	-0.886
c vs p								
PPO9	**2.03**	**5.01**	**4.30**	3.78	1.56	1.10	**1.92**	0.49
a vs f								
PPO9	**1.38**	**3.76**	**3.24**	2.79	1.25	0.89	**1.48**	-0.33
a/p vs f								
PPO9	**0.38**	**0.22**	**0.24**	0.28	0.09	0.06	**-1.84**	-0.56
m vs f								
TEP15	**4.34**	**4.06**	**5.19**	4.53	0.59	0.42	**2.18**	-0.22
c vs p								
TEP15	**0.40**	**0.52**	**0.30**	0.41	0.11	0.08	**-1.30**	-1.287

a vs f								
TEP15	**0.81**	**2.50**	**2.36**	1.89	0.94	0.67	**0.92**	0.744
a/p vs f								
TEP15	**1.27**	**1.10**	**1.11**	1.16	0.10	0.07	**0.21**	0.263
m vs f								
AgMDL1	**1.97**	**1.02**	**1.50**	1.50	0.48	0.34	**0.58**	0.081
c vs p								
AgMDL1	**1.53**	**1.59**	**1.44**	1.52	0.08	0.05	**0.60**	0.573
a vs f								
AgMDL1	**0.99**	**2.79**	**1.82**	1.87	0.90	0.64	**0.90**	0.302
a/p vs f								
AgMDL1	**1.13**	**1.19**	**1.59**	1.30	0.25	0.18	**0.38**	0.49
m vs f								
BRP	**6.42**	**4.11**	**7.19**	5.91	1.60	1.14	**2.56**	0.86
c vs p								
BRP	**2.75**	**3.54**	**3.55**	3.28	0.46	0.32	**1.71**	1.103
a vs f								
BRP	**1.42**	**2.51**	**3.36**	2.43	0.97	0.69	**1.28**	0.487
a/p vs f								
BRP	**2.40**	**1.98**	**3.06**	2.48	0.54	0.39	**1.31**	0.189
m vs f								
TEP4	**0.37**	**0.56**	**0.65**	0.53	0.14	0.10	**-0.93**	-0.28
c vs p								
TEP4	**0.33**	**0.27**	**0.37**	0.32	0.05	0.04	**-1.63**	-1.548
a vs f								
TEP4	**3.70**	**4.23**	**4.07**	4.00	0.27	0.19	**2.00**	0.89
a/p vs f								
TEP4	**2.33**	**3.47**	**1.59**	2.46	0.95	0.67	**1.30**	0.42

m vs f								
GNBPB3	**2.32**	**3.09**	**3.37**	2.93	0.54	0.39	**1.55**	0.423
c vs p								
GNBPB3	**0.44**	**0.38**	**0.39**	0.40	0.03	0.02	**-1.31**	-0.786
a vs f								
GNBPB3	**1.64**	**1.70**	**1.61**	1.65	0.05	0.03	**0.72**	0.236
a/p vs f								
GNBPB3	**1.42**	**1.46**	**1.17**	1.35	0.16	0.11	**0.43**	0.636
m vs f								
kininogen	**2.73**	**3.19**	**2.54**	2.82	0.33	0.24	**1.50**	0.207
c vs p								
kininogen	**2.70**	**2.38**	**2.57**	2.55	0.16	0.11	**1.35**	1.162
a vs f								
kininogen	**1.59**	**2.06**	**2.81**	2.15	0.62	0.44	**1.11**	0.372
a/p vs f								
kininogen	**1.41**	**1.17**	**1.49**	1.36	0.17	0.12	**0.44**	0.138
